# Omega-3 Fatty Acids Supplementation Differentially Modulates the SDF-1/CXCR-4 Cell Homing Axis in Hypertensive and Normotensive Rats

**DOI:** 10.3390/nu9080826

**Published:** 2017-08-01

**Authors:** Luiza Halmenschlager, Alexandre Machado Lehnen, Aline Marcadenti, Melissa Medeiros Markoski

**Affiliations:** 1 Postgraduate Program in Health Sciences: Cardiology, Institute of Cardiology of Rio Grande do Sul/University Foundation of Cardiology (IC/FUC), Princesa Isabel Avenue, 370, Porto Alegre, RS 90620-001, Brazil; luizahal@hotmail.com (L.H.); amlehnen@gmail.com (A.M.L.); alinemo@ufcspa.edu.br (A.M.); 2 Laboratory of Biodynamics, Sogipa School of Physical Education, Benjamin Constant Avenue, 80, Porto Alegre RS 90550-003, Brazil; 3 Postgraduate Program in Nutrition Sciences, Federal University of Health Sciences of Porto Alegre (UFCSPA), Sarmento Leite Avenue, 245, Porto Alegre RS 90050-170, Brazil

**Keywords:** ω-3 fatty acid, spontaneously hypertensive rats, Wistar Kyoto rats, cell homing, hypertension, Stromal Derived Factor-1, CXCR4 receptor

## Abstract

Background: We assessed the effect of acute and chronic dietary supplementation of ω-3 on lipid metabolism and cardiac regeneration, through its influence on the Stromal Derived Factor-1 (SDF-1) and its receptor (CXCR4) axis in normotensive and hypertensive rats. Methods: Male Wistar Kyoto (WKY) and spontaneously hypertensive rats (SHR) were allocated in eight groups (of eight animals each), which received daily orogastric administration of ω-3 (1 g) for 24 h, 72 h or 2 weeks. Blood samples were collected for the analysis of the lipid profile and SDF-1 systemic levels (ELISA). At the end of the treatment period, cardiac tissue was collected for CXCR4 expression analysis (Western blot). Results: The use of ω-3 caused a reduction in total cholesterol levels (*p* = 0.044), and acutely activated the SDF-1/CXCR4 axis in normotensive animals (*p* = 0.037). In the presence of the ω-3, after 72 h, SDF-1 levels decreased in WKY and increased in SHR (*p* = 0.017), and tissue expression of the receptor CXCR4 was higher in WKY than in SHR (*p* = 0.001). Conclusion: The ω-3 fatty acid supplementation differentially modulates cell homing mediators in normotensive and hypertensive animals. While WKY rats respond acutely to omega-3 supplementation, showing increased release of SDF-1 and CXCR4, SHR exhibit a weaker, delayed response.

## 1. Introduction

Hypertension (HTN) is the leading cause of cardiovascular disease [1] and strongly contributes to worldwide mortality [2]. Although dietary interventions have been related to the control of blood pressure levels [3,4] and reduced incidence of cardiovascular disease [5], all possible underlying molecular and cellular mechanisms involved in these interactions are still unknown.

The World Health Organization (WHO)’s guidelines on the prevention of cardiovascular diseases [6] point out that the consumption of fish and fish oils is associated with decreased cardiovascular risks, something which is also defended by the so-called Mediterranean Diet (MeDiet) [7]. In this way, the intake of marine omega-3 (ω-3) polyunsaturated fatty acids (PUFA), EPA (eicosapentaenoic) and DHA (docosahexaenoic) acids, has been related to the prevention of heart disease by a variety of mechanisms, including management of atrial fibrillation [8], blood pressure control [9,10], modulation of the lipid profile [11], and coronary heart disease prevention [12,13]. Such effects of ω-3 PUFA can be related to their anti-inflammatory activities, which inhibit the release of pro-inflammatory cytokines and the formation of platelets [14,15]. In addition, ω-3 PUFA influence eicosanoid metabolism, nuclear factor kappa B (NF-κB) gene expression, intercellular communication, cell membrane phospholipid fatty acid composition, which also depends on the amount of dietary PUFA intake [16]. Despite all benefits, the role of ω-3 PUFA in regenerative medicine, especially in the process of stem cell homing, including migration, proliferation, differentiation, and engrafting of cells, is unknown.

Stem cells are able to multiply while maintaining their undifferentiated state (self-renewal capability) and actively replacing damaged cells in tissues, and may also differentiate into various cell types. Therefore, it is believed that adult stem cells, present in different tissues, have a regenerative role when they are exposed to injury [17,18]. The stromal cell-derived factor-1 (SDF-1), a chemokine secreted in situations of tissue stress, and its receptor CXCR4 (CXC-Chemokine Receptor Type 4), anchored to the outer membrane of stem cells and some immune system cells, are the main elements involved in cell homing [19].

The expression and release of SDF-1 by damaged tissue acts as a positive allosteric modulator, promoting the migration of progenitor cells from the bone marrow and different organs towards the SDF-1 gradient, and hence to the site of the lesion [20]. Thus, physiologically, the SDF-1/CXCR-4 axis is also responsible for organogenesis and replacement of apoptotic or senescent cells in healthy tissues [21]. Both processes can be influenced by eating habits.

The aim of this study was to investigate the effects of ω-3 PUFA supplementation on cell homing, precisely on the expression of SDF-1 and its receptor CXCR4 in spontaneously hypertensive rats (SHR) and normotensive Wistar-Kyoto (WKY) rats in different time intervals, to test the acute, subacute and the chronic effects of the supplemented diet.

## 2. Materials and Methods

All procedures were carried out according to the National Institute of Health Guide for the Care and Use of Laboratory Animals [22] and to the Brazilian College of Animal Experimentation (COBEA). This study was approved by the Committee of Ethics of Instituto de Cardiologia do Rio Grande do Sul (protocol UP4503/10).

### 2.1. Sample Groups, Treatments and Blood Collection

Male WKY and SHR aged 90 days were used in this study. The animals were obtained from laboratory animal house of Fundacao Estadual de Producao em Pesquisa em Saude (FEPPS), Porto Alegre, Brazil. The average values of pressure for these animals, at 3 months old, were 123.16 ± 12.86 mmHg (WKY) and 183.50 ± 14.27 (SHR) in the baseline and 120.20 ± 11.51 mmHg (WKY) and 179.80 ± 13.91 mmHg (SHR) at the end of 2 weeks (for groups that receive ω-3). The rats were kept in plastic cages with wire floors, at 22–24 °C, 12 h light/dark cycle, and fed ad libitum with water and a commercial rat chow (Nuvilab, Colombo, Brazil) containing 19.0% of protein, 56.0% of carbohydrate, 3.5% of lipids, 4.5% cellulose, 5.0% of vitamins and minerals and 17.03 kJ/g of energy. Rats were treated with 1 g/day of marine ω-3 (fish oil), containing 180 mg of EPA and 120 mg of DHA by gastric gavage. The administrations were carried out early in the morning and blood collection and/or euthanasia occurred 24 h (acute effect), 72 h (subacute effect) or 14 days (chronic effect) after. The marine ω-3 was acquired commercially (Confiare, Porto Alegre, Brazil) in form of gelatin capsules (free from any microbe contamination) that were aseptically opened for oil removing on each day of administration. The dosage is safe for rats [23] and compatible with the recommended amount of fish oil for humans [24]. For both animal models (WKY and SHR), we used 8 animals per group, distributed in: control group (Gc), animals that received only water (1 mL) by gastric gavage; 24 h group (G24h); 72 h group (G72h); and 2 weeks group (G2w). All animals were euthanized immediately after the treatment period (24 h, 72 h or 2 weeks).

The animals were weighed every day and subjected to blood collection (100 µL, by puncture of tail vein) at baseline, under anesthesia with 0.2 mL/100 g of ketamine (50 mL/kg and xylazine (20 mL/kg), and at the moment of euthanasia (2 mL by cardiac puncture), also under anesthesia (same as described above). The G2w rats also had a blood collection in the middle of treatment, after 1 week of fatty acid administration. This sample and the baseline sample of the control group were respectively named “G1w” and “Baseline”. The biological tests were carried out according to the Guide for the Care and Use of Laboratory Animals [22] and the Brazilian legislation (law number 11794) for the care and use of laboratory animals.

### 2.2. Recovery of Biological Materials

All blood samples were centrifuged at 2000 rpm for 10 min, and the plasma were aliquoted and stored at −20 °C until use. After euthanasia, the hearts were removed, immediately weighed, placed into cryogenic tubes and immersed in liquid nitrogen. After freezing, the samples were transferred and stored at −80 °C. The tissues were homogenized in 5 mL of buffer (pH 7.4, 0.6057 mmol/L Tris-base, Invitrogen; 0.18612 mmol/L Ethylenediamine tetraacetic acid (EDTA), Invitrogen; and 42.79 mmol/L sucrose, Synth), using a mechanical homogenizer (Polytron, Marconi, Piracicaba, Brazil), as described by Mori et al. (2008) [25]. The homogenized samples were transferred to 50 mL tubes and centrifuged at 1700 rpm, for 10 min at 4 °C. The supernatant (~3 mL), containing total protein extracts, was collected and stored at −20 °C until use.

### 2.3. Biochemical Analysis of Metabolic Markers

Concentrations of total cholesterol (COL), high-density lipoproteins (HDL-cholesterol) and triglycerides (TGL) were determined by colorimetric assay using commercial kits (Labtest Diagnostica SA, Lagoa Santa, Brazil). Optical densities were measured by spectrophotometry (Spectramax M2e, Molecular Devices, Sunnyvale, CA, USA) at 500–505 nm. Baseline measurements were obtained by comparing the optical densities of the samples with the respective standards, available in the kits. Data were expressed in milligrams per deciliter (mg/dL).

### 2.4. ELISA and Western Blot Analysis

Systemic levels of SDF-1α were determined by enzyme-linked immunosorbent assay (ELISA) using a commercial kit (Cusabio, Wuhan, China), in accordance with the manufacturer’s instructions. The optical densities were measured in a spectrophotometer (Spectramax M2e) at 450 nm and 25 °C, with background subtraction at 570 nm. Baseline measurements were obtained by linear regression of the 4 parameters. Data were expressed in picograms of protein per milliliter (pg/mL).

Protein concentration of the samples was determined by the Bradford method [26]. Samples containing 100 µg of total protein extract were mixed with NuPage transfer buffer (Invitrogen, Carlsbad, CA, USA), denatured by incubation at 100 °C for 5 min, and separated on a 12% denaturing polyacrylamide gel electrophoresis. Later, proteins were transferred to nitrocellulose membrane Hybond ECL (GE Healthcare, Cleveland, OH, USA) using a semi-dry system (Amersham Biosciences, Little Chalfont, UK), in the same buffer with 20% methanol (Merck, Kenilworth, NJ, USA), at 100 mA for 3 h at room temperature. After the transfer, the membranes were stained with Ponceau, photographed and washed in phosphate-buffered saline (1X PBS) to remove the dye. The membranes were blocked with non-fat dried milk, and subjected to immunodetection using anti-CXCR4 antibody (Santa Cruz Biotech, Dallas, TX, USA). The membranes were incubated with 100 mg of secondary antibody (anti-rabbit IgG, Millipore, Billerica, MA, USA; titration of 1:5000) diluted in 20 mL of 5% casein solution for 16 h at 4 °C, followed by an additional incubation for 3 h at 37 °C under agitation. For chemiluminescence detection, the membranes were incubated for 3 min with a solution containing hydrogen peroxide and luminol (ECL kit, GE Healthcare, Cleveland, OH, USA). The membranes were then exposed to X-ray film (Ge Healthcare, Cleveland, OH, USA) for 1 min, 5 min, 15 min, 30 min or 2 h in the dark. The films were then scanned and quantified by optical densitometry with the software Scion Image (Scion Corporation, Frederick, MD, USA). The results were expressed as *arbitrary units* (AU) and related to the total sample volume, weight of the tissue and weight of the animal.

### 2.5. Statistical Analysis

The normality of the variables’ distribution was analyzed by the Shapiro-Wilk test. Data with normal distribution were represented as means and standard deviation, and those with non-normal distribution as median and interquartile range. The differences among groups were tested using Kruskall Wallis with Student–Newman-Keuls post-test (non-normal distribution variables) or a generalized estimating equation (GEE) followed by Bonferroni’s post-hoc test (normal distribution variable). Correlations were analyzed by Spearman’s rank correlation. The significance level used for all tests was 5%. Analyses were performed using the software BioEstat version 5.3 [27] and the Statistical Package for the Social Sciences (SPSS) version 23 (IBM).

## 3. Results

### 3.1. Omega-3 Supplementation Does not Cause Changes in Body Weight but Modifies the Lipid Profile

**Figure 1:**
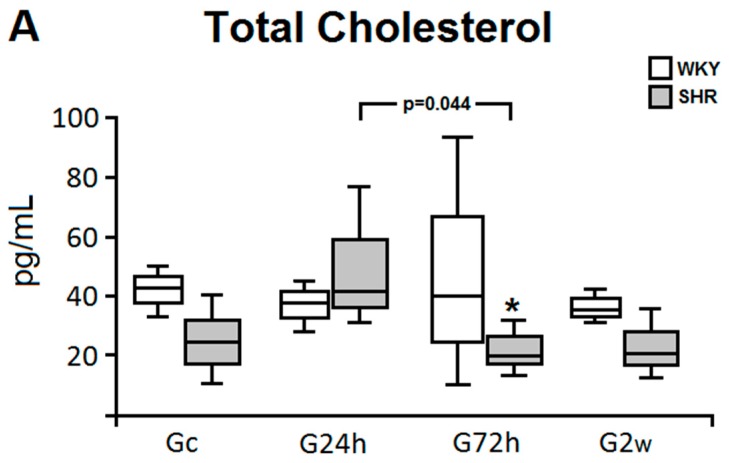

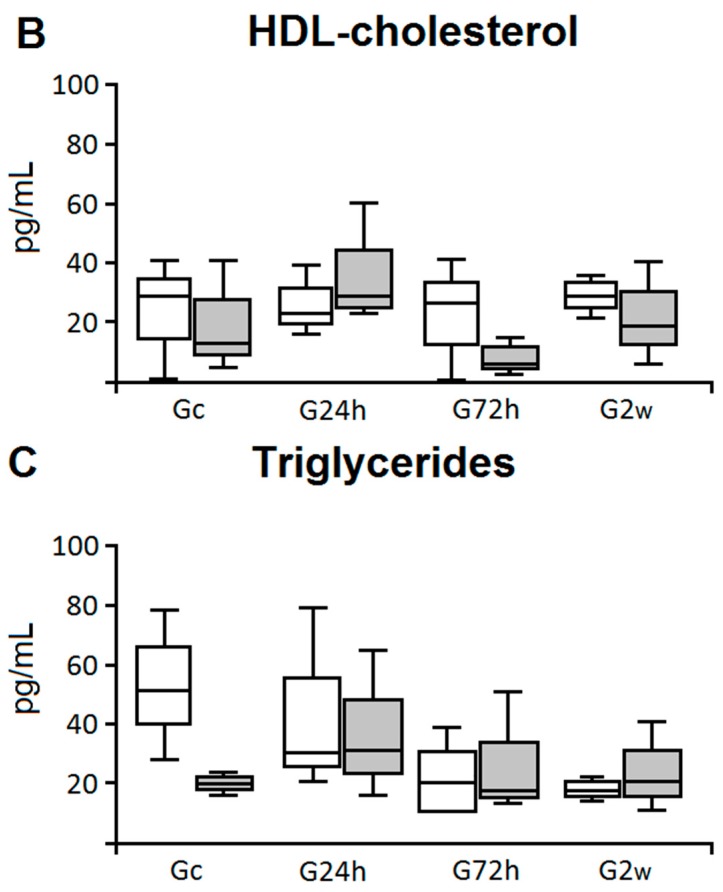
Lipid profile of normotensive and hypertensive rats after daily supplementation with omega-3 (ω-3). The levels of total cholesterol (**A**), cholesterol-HDL (**B**) and triglycerides (**C**) were quantified after 24 h, 72 h and 2 weeks of ω-3 supplementation and after 2 weeks of water intake in normotensive (WKY) and hypertensive (SHR) animals. The *p* values for comparisons between models at a specific time are shown in the panel. * *p* = 0.001, G72h SHR vs. G72h WKY. HDL, High-Density Lipoprotein; Gc, Control group; G24h, 24 h group; G72h, 72 h group; G2w, 2 weeks group.

After two weeks of treatment, the effect of ω-3 PUFA supplementation on body weight was compared between the WKY and SHR (Table 1), and no significant changes were observed in both groups. The initial and final body weights (WKY and SHR) were 268.97 ± 26.22 g and 283.03 ± 22.68 g, respectively (*p* = 0.073). Nevertheless, daily ω-3 PUFA supplementation caused reduction in COL levels in SHR animals after 72 h of treatment, as compared with the G24h (*p* = 0.044), and WKY rats (*p* = 0.001) (Figure 1A). Although changes in triglycerides and HDL-cholesterol levels were also observed (Figure 1B,C), they were not statistically significant.

### 3.2. ω-3 PUFA Reduced the Release of SDF-1 in Normotensive Rats and Increased in Hypertensive Rats

**Figure 2:**
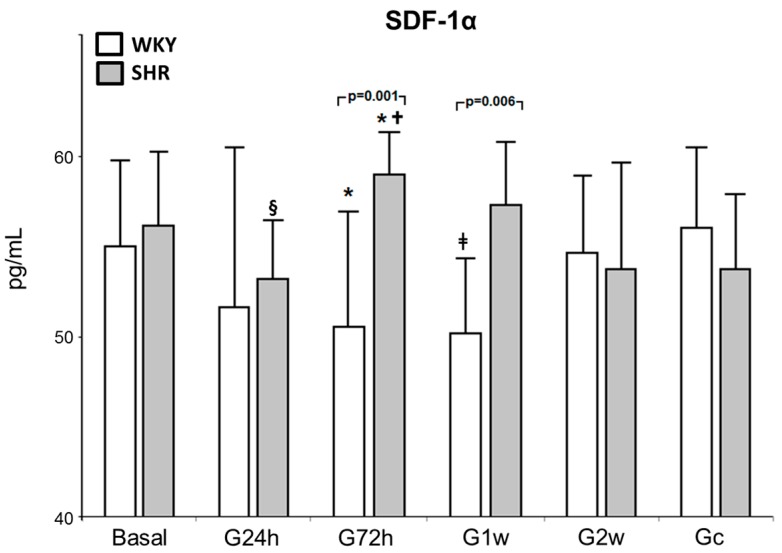
Systemic release of SDF-1 in normotensive and hypertensive rats after daily supplementation with ω-3. Normotensive (WKY) and hypertensive (SHR) animals had plasma collected in basal period and after 24 h, 72 h, 1 and 2 weeks of ω-3 supplementation and after 2 weeks of water intake. Data are expressed in picograms/milliliter (pg/mL). The *p* values for comparisons between models at a specific time are shown in the panel. * *p* < 0.05 vs. Gc; ^§^ *p* < 0.05 vs. G2w; ^†^ *p* < 0.05 vs. G24h; ^‡^ *p* < 0.05 vs. Basal. SDF-1, Stromal-Derived Factor-1; Gc, Control group; G24h, 24 h group; G72h, 72 h group; G1w, 1 week group; G2w, 2 weeks group.

Significant differences in SDF-1α concentrations were found between treatment groups (*p* = 0.017) (Figure 2). The animal models showed different behaviors in relation to cytokine release in response to ω-3 PUFA supplementation: WKY rats showed a greater reduction in SDF-1α release when compared to SHR rats at 72 h (16.8%, *p* = 0.001) and after one week (14.3%, *p* = 0.006) of treatment. When the period of ω-3 PUFA supplementation was analyzed by group, a decrease in SDF-1 release was detected in WKY rats after 24 h (55–51 pg/mL), and remained decreased after one week, and returned to basal levels after two weeks (50–55 pg/mL) with similar concentrations of those in the Gc (56 pg/mL). On the other hand, SHR rats showed a small increase in SDF-1 levels after 72 h of supplementation (56–59 pg/mL), similar to the control group, but this effect was not maintained after the two-week period (54 pg/mL) (Figure 2).

### 3.3. CXCR4 Expression in Cardiac Tissue Was Differentially Modulated by ω-3 PUFA Supplementation in Normotensive and Hypertensive Rats

**Figure 3:**
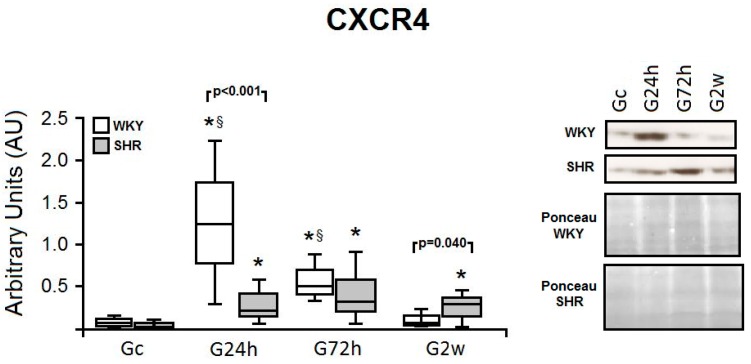
Cardiac tissue expression of CXCR4 in normotensive and hypertensive rats after daily supplementation with ω-3. Normotensive (WKY) and hypertensive (SHR) animals were submitted to CXCR-4 protein analysis in the heart tissue after 24 h, 72 h and 2 weeks of ω-3 supplementation and after 2 weeks of water intake. Data obtained by densitometry were compared to heart and animal weight, ponceau staining and are expressed in Arbitrary Units (AU). The right panel shows representative *blots* and reference bands (stained with Ponceau red) for all groups. The *p* values for comparisons between models at a specific time are shown in the figure. * *p* < 0.05 vs. Gc; ^§^ *p* < 0.05 vs. G2w. CXCR4, C-X-C chemokine receptor type 4; Gc, Control group; G24h, 24 h group; G72h, 72 h group; G2w, 2 weeks group.

The expression of the CXCR4 receptor was compared between WKY and SHR throughout the two-week treatment period (Figure 3). Normotensive rats showed high expression of the receptor in the early period (G24h vs. Gc, *p* = 0.001), which returned to basal levels at the end of the protocol (G24h vs. G2w, *p* = 0.005). In contrast, SHR showed a significant, gradual increase in CXCR4 expression in cardiac tissue as compared with the Gc, which remained increased after two weeks (G24h, *p* = 0.003; G72h, *p* = 0.016; G2w, *p* = 0.014). CXCR4 expression was higher in SHR than in WKY animals in both acute (*p* = 0.001) and chronic period (*p* = 0.04). Thus, SDF-1 receptor expression is differently modulated by ω-3 PUFA supplementation in the heart tissue of SHR and WKY rats.

### 3.4. Activation of the SDF-1/CXCR-4 Axis Was Influenced by Acute Administration of ω-3 PUFA and Was Not Dependent on Changes of Cardiovascular Dynamics

**Figure 4:**
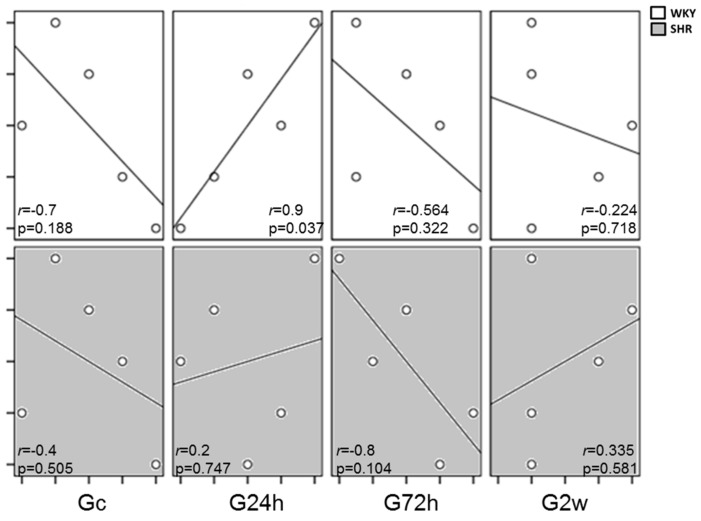
Correlations between the SDF-1 release and the tissue expression of CXCR-4 in normotensive and hypertensive rats after daily supplementation with ω-3. The normotensive (WKY) and hypertensive (SHR) animals were compared as the SDF-1/CXCR-4 axis activation during the time interval of dietary supplementation with ω-3. *r* = Spearman’s coefficient. The *p* values are indicated in the panel. Gc, Control group; G24h, 24 h group; G72h, 72 h group; G2w, 2 weeks group.

To analyze the influence of PUFA and blood pressure on the activation of the SDF-1/CXCR4 axis, we compared the results of the systemic release of SDF-1 and the cardiac tissue expression of its receptor between the animal models. The WKY rats showed a high correlation (*r* = 0.9; *p* = 0.037) between these parameters after the first 24 h of ω-3 PUFA supplementation (Figure 4). These results show that ω-3 PUFA supplementation had an acute, transient effect on the activation of homing molecules in normotensive animals.

## 4. Discussion

The present study showed the effects of dietary supplementation with ω-3 PUFA on cell homing in the setting of HTN, compared with normal blood pressure. Here, we noticed that ω-3 PUFA acutely induced CXCR4 expression in the cardiac tissue of normotensive animals (24 h after supplementation). In SHR rats, despite increased release of the ligand SDF-1 in response to ω-3 PUFA supplementation, only a small increase on the receptor expression was detected after 72 h of treatment.

The supplementation with ω-3 PUFA did not promote weight gain, which reflected a pattern of weight gain expected for healthy young rats. Indeed, it has been postulated that diets with high contents of marine ω-3 PUFA may decrease fat synthesis, contribute to body fat reduction, and be used for the treatment of obesity [28]. Also, ω-3 PUFA may positively influence the immune system and reduce low-grade inflammation [29,30]. Moreover, the control groups were treated with water instead of other vehicles to ensure that the control supplementation was inert on the activation of any biochemical mechanism or weight gain.

It was also reported that ω-3 PUFA modulates the expression of genes involved in lipid metabolism and adipogenesis, acting as ligand to important transcription factors, such as the peroxisome proliferator-activated receptors (PPAR) [16]. In this context, we also evaluated the influence of ω-3 PUFA on lipid profile. The supplementation with ω-3 PUFA had a positive effect in reducing COL levels in hypertensive animals after 72 h of supplementation. Since we did not detect important reductions in HDL-cholesterol levels in both groups, the reduction in COL levels may have been due to a decrease in low density lipoprotein (LDL-cholesterol) levels, which were not measured in this study. Many hypotheses about the mechanism by which PUFA decrease blood cholesterol levels have been considered, by increasing the formation of bile acid promoting a redistribution of cholesterol in the tissues, and by increasing LDL receptors in the liver, leading to a decrease in cholesterol plasma concentrations [31]. Lombardo et al. (2013) [32] have indicated that ω-3 PUFA have an effect in cholesterol reduction, which corroborates the fact that the decrease in COL levels in both normotensive and hypertensive rats in our study was due to the ω-3 PUFA supplementation. However, it is important to mention that the SHR model presents polymorphisms in the gene that encodes the epoxide hydrolase (EPHX2), an enzyme related to renal metabolism of arachidonic acid, transient states of anorexia and imbalances in cholesterolemic levels after ingestion of fatty acids. We believe this slight initial increase (after ingestion of omega-3) could be resultant of an action of this and its consequent regulation [33]. Studies that evaluated the effect of EPA and DHA on the lipid profile have shown, in general, a reduction of LDL-cholesterol [34], triglyceridemia [35], apolipoproteins, and an increase in lipoprotein lipase activity [36], an enzyme that hydrolyzes triglycerides. Lipid and lipoprotein metabolism changes significantly with the regular consumption of fish or nutritional supplementation with marine ω-3 PUFA, and doses lower than 2 g/day are sufficient to produce such effects [37]. In addition, Colussi et al. (2004) showed that ω-3 PUFA administered to hypertensive subjects (1 g/day by 6 months or 4 g/day by 1 month) were able to decrease plasma levels of Lipoprotein (a), a similar LDL particle identified as a risk factor for atherosclerotic disease [38]. Therefore, we believe that the amounts of ω-3 PUFA used in this study were able, at least in part, to positively influence the metabolism of lipids, which, for humans, may be beneficial in the prevention and treatment of HTN and other cardiovascular risk factors.

Regarding the effect of ω-3 PUFA supplementation on cell homing in normotensive and hypertensive animals, the intervention induced a pronounced increase in CXCR4 expression in the cardiac tissue and a decrease in systemic SDF-1 levels in WKY animals in the acute phase, possibly due to recruitment of the ligand by CXCR4^+^ cells. However, such effect was not sustained in the chronic phase, maybe due to the absence of cooperative signaling from inflammation, oxidative stress and hypoxia response. The opposite occurred in the SHR, who showed a signaling response that promotes the expression of cytokines in response to the injury [39] and enhances cell homing, mainly during the chronic phase of ω-3 PUFA supplementation. In fact, marine ω-3 PUFA activates PPAR [40], that triggers the immune response, as well as chronic inflammatory cytokines, reactive oxygen species and transcription factors like the hypoxia inducible factor 1 (HIF-1) and NF-kB, which lead to the activation of SDF-1. Both DHA and EPA also exert anti-inflammatory properties [41], allowing a balance between anti-inflammatory molecules and pro-inflammatory cytokines involved in cell homing.

The chemokine SDF-1 is primarily expressed in high levels by bone marrow stromal cells [42] and several studies have been carried out to evaluate the action of this ligand and its receptor CXCR4 on tissue regeneration [43,44,45], acting in both physiological cell replacement and also under lesion or injury [45,46,47]. In cardiac tissue, the increase in SDF-1 levels stimulates the recruitment of cells to the site of injury (niche), which promote tissue repair and display positive paracrine effects on cardiomyocyte survival and cardiac function [48,49,50]. Our study is the first to show the relationship between HTN and the activity of SDF-1/CXCR4 axis that may be modulated by functional nutrients. It is worth mentioning that neither the cytokine levels nor its receptor expression were changed in the control group, which received only water.

Recently, it has been reported that the SDF-1 is an important regulator of the sympathetic nervous system and hemodynamic function in normal or pathological conditions, and it may contribute to neural and humoral activation in heart failure [51], the main pathological consequence of HTN. The ω-3 PUFA was able to sustain the expression of this molecule during the entire period of protocol in hypertensive animals, probably through the maintenance of common signaling pathways that lead to the release of SDF-1 as inflammatory cytokines. Although the dietary intake of ω-3 PUFA in the form of food-sources like fish oil has no direct effect on the treatment to cardiovascular diseases [52], it may be related to the secondary prevention of heart failure [53,54]. However, the mechanisms involved in the effects of cardioprotective substances or functional foods on the homing of stem cells still require further investigations. We found a differential influence of the ω-3 fatty acid from fish oil on cell homing, mediated by an acute modulation of the SDF-1/CXCR4 axis activity in normotensive animals, and a late response in those with altered cardiovascular dynamics as in HTN. Additional studies with different experimental models are needed for a better understanding of specific mechanisms and mediators involved in cell homing in cardiovascular disease. This information can be used to develop targeted interventions involving nutritional factors aimed at the prevention and treatment of this condition.

## 5. Conclusions

This study shows that omega-3 polyunsaturated fatty acids can modulate molecules involved in cell homing in a time-dependent manner and according to blood pressure conditions. While normotensive animals respond acutely (72 h) to omega-3 supplementation, showing increased release of the chemokine SDF-1 and its receptor CXCR4, hypertensive rats exhibit a weaker, delayed response. Understanding how functional foods can affect cell response and their contributions to prevention/regulation of HTN can support effective therapeutic interventions. Additionally, clarifying the mechanisms that regulate stem-cell homing may help the management of cell therapy protocols, not only for cardiovascular diseases, but also for other conditions involving tissue regeneration and repair.

## Figures and Tables

**Table 1:** Body weight of animals that received supplementation with water (control) or ω-3 in the initial phase and ending of the treatment (2 weeks).

Model	Treatment	Initial Weight (g)	Final Weight (g)	*p*-Value
WKY	water	274.38 ± 12.35	297.00 ± 16.00	0.116
WKY	ω-3	232.63 ± 33.51	250.25 ± 30.40	0.221
SHR	water	273.63 ± 46.24	285.50 ± 49.49	0.410
SHR	ω-3	295.25 ± 16.20	299.38 ± 16.78	0.775

WKY, Wistar-Kyoto rats; SHR, spontaneously hypertensive rats.
